# Protecting the LIMA During Scoring Balloon Delivery to the LAD Using an Integrated Microcatheter-Guide Extension

**DOI:** 10.1016/j.jaccas.2026.108966

**Published:** 2026-07-02

**Authors:** Asrar Abdou, Asaad Nakhle, Vikas Aggarwal, Mohammad Alqarqaz, Gerald C. Koenig, Brittany S. Fuller, Herbert Aronow, Khaldoon Alaswad

**Affiliations:** a Division of Cardiology, Henry Ford Hospital, Detroit, Michigan, USA; b Wayne State University, School of Medicine, Detroit, Michigan, USA; c Michigan State University, College of Human Medicine, East Lansing, Michigan, USA

**Keywords:** atherosclerosis, cardiovascular disease, coronary artery bypass, lifestyle, myocardial revascularization, percutaneous coronary intervention

## Abstract

**Background:**

Percutaneous coronary intervention via the left internal mammary artery (LIMA) graft is technically challenging and carries a risk of graft injury, particularly during delivery of specialty balloons.

**Case Summary:**

A 40-year-old man with premature coronary artery disease and prior coronary artery bypass grafting 17 years ago presented with refractory angina. Coronary angiography demonstrated severe left anterior descending artery stenosis distal to the LIMA anastomosis. After inadequate lesion expansion, scoring balloon was required. To minimize the risk of LIMA graft trauma during device delivery, an integrated microcatheter-guide extension system was advanced into the distal LIMA, allowing safe delivery of the scoring balloon followed by stenting with excellent angiographic results.

**Discussion:**

This case highlights a novel strategy to protect the LIMA graft during high-risk device delivery, using an integrated microcatheter-guide extension system.

**Take-Home Message:**

Integrated microcatheter-guide extension systems may reduce the risk of LIMA graft injury and facilitate delivery of specialty balloons in complex percutaneous coronary intervention.

## Visual Summary

**Figure:**
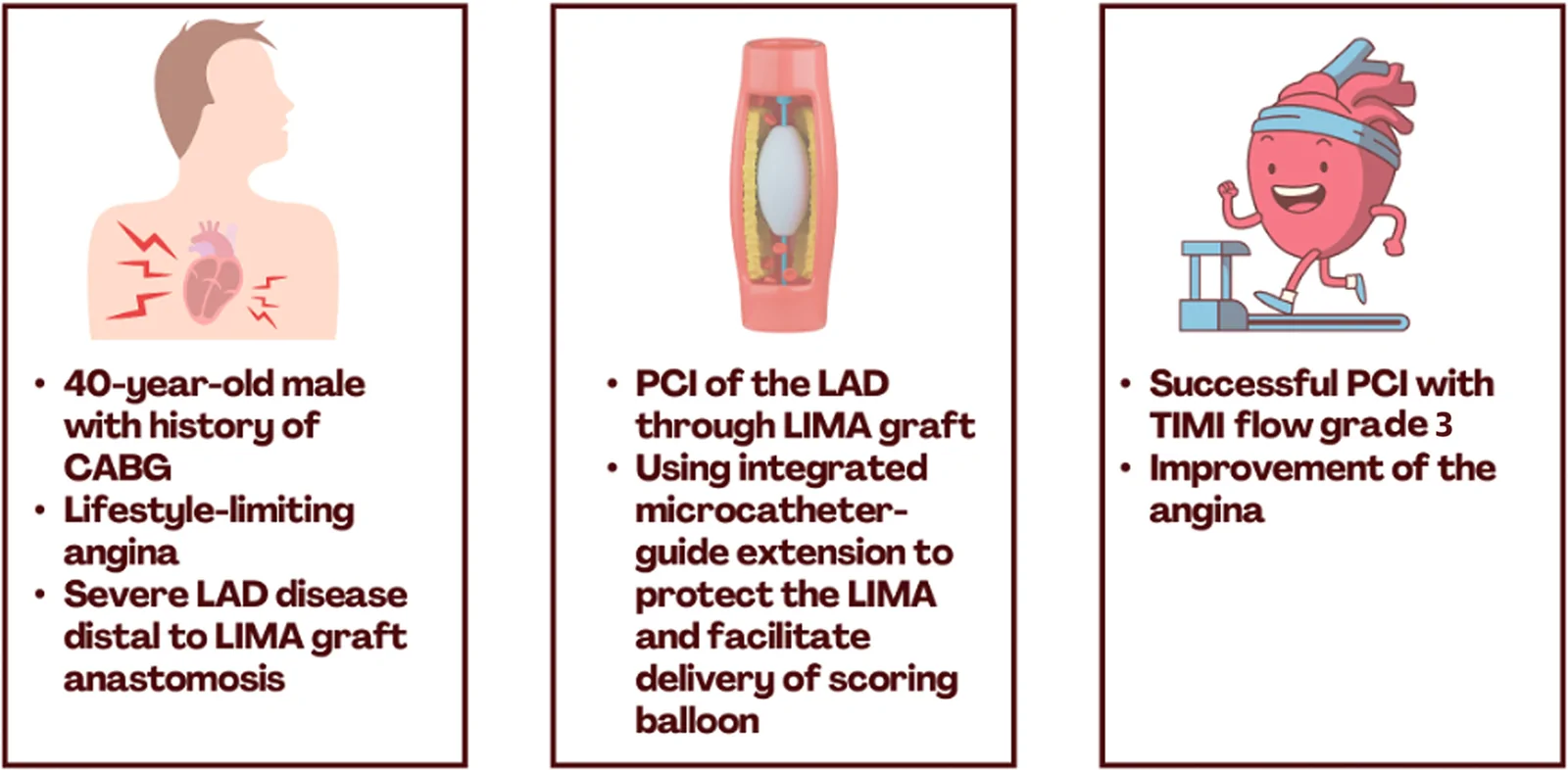

**Visual Summary:**
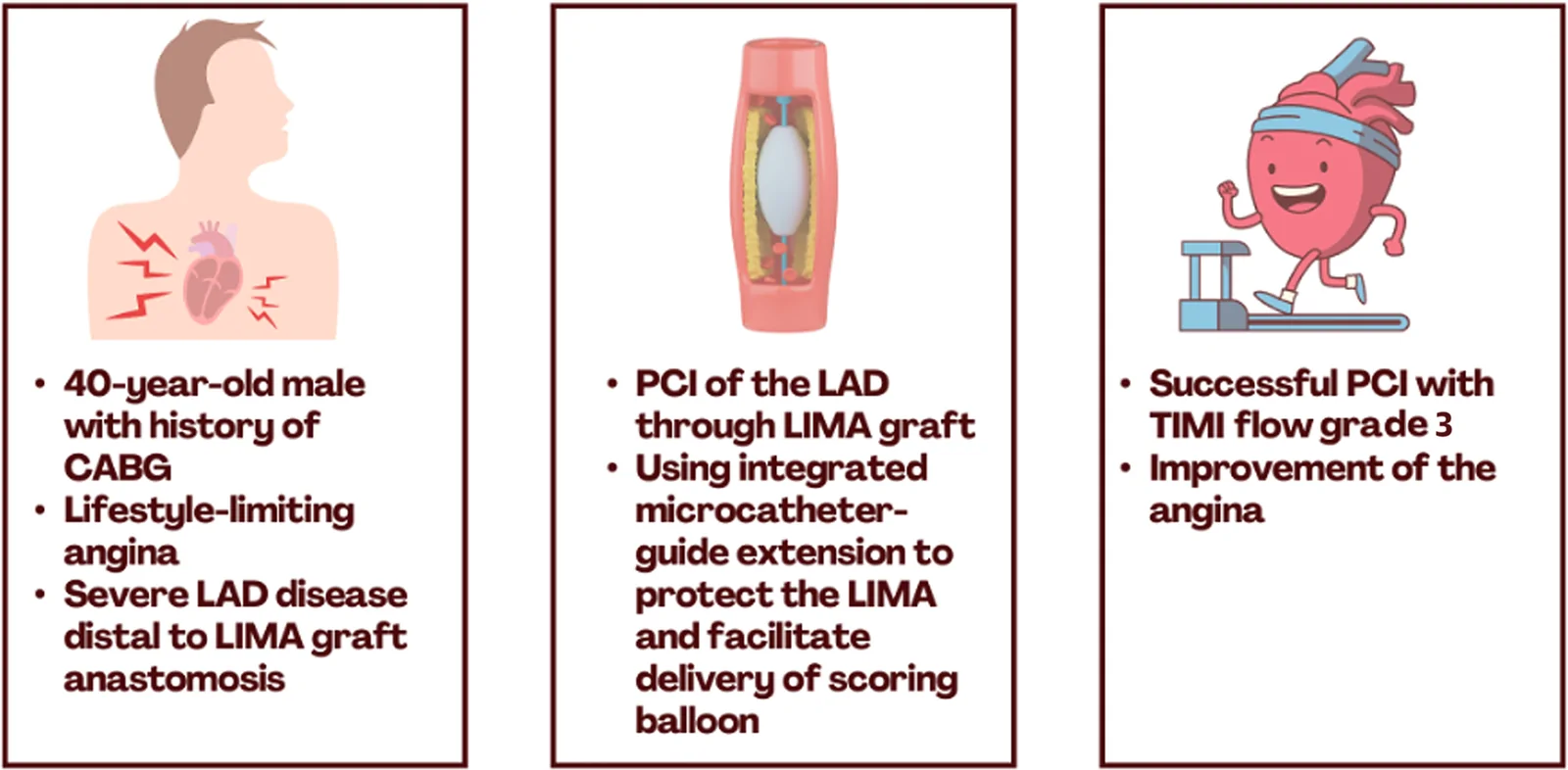
Protecting the LIMA During Scoring Balloon Delivery to the LAD Using an Integrated Microcatheter-Guide Extension CABG = coronary artery bypass graft; LAD = left anterior descending artery; LIMA = left internal mammary artery; PCI = percutaneous coronary intervention.

## History of Presentation

A 40-year-old man presented with lifestyle-limiting angina refractory to optimal medical therapy, including long-acting nitrate, beta-blocker, and calcium channel blocker treatment. His symptoms were consistently provoked by exertional activities such as climbing on flight of stairs. He was hemodynamically stable, and physical examination was unremarkable, with no signs of heart failure or other acute pathology.

## Past Medical History

His medical history was significant for hypertension, type 2 diabetes mellitus, severe primary hypercholesterolemia with markedly elevated lipoprotein(a), and premature severe coronary artery disease. He sustained a myocardial infarction in July 2009 and subsequently underwent coronary artery bypass grafting with the left internal mammary artery (LIMA) to the left anterior descending artery (LAD), the right internal mammary artery to the right coronary artery, and a radial artery graft to the ramus intermedius and diagonal branch arteries.

## Investigations

Coronary angiography demonstrated a patent LIMA graft to the LAD with severe (approximately 80%) stenosis of the native LAD, distal to the LIMA anastomosis. The right internal mammary artery graft to the right coronary artery and the radial artery graft to the obtuse marginal and ramus branch arteries were patent. Severe stenosis of the native posterior descending artery (PDA) was also appreciated distal to the graft anastomosis (Video 1, Video 2, Video 3). Given the patient’s symptoms and angiographic findings, a decision was made to proceed with percutaneous coronary intervention (PCI) of the LAD lesion via the LIMA graft, with staged PCI of the PDA if symptoms remained lifestyle limiting.

## Differential Diagnosis

The differential diagnosis for the patient’s refractory angina included progression of native coronary artery disease distal to the bypass graft anastomosis, graft failure or competitive flow-related ischemia, new de novo disease in an unbypassed native coronary artery, and microvascular angina. In the context of the patient’s symptom profile and angiographic findings, the severe focal stenosis of the LAD distal to the LIMA anastomosis was considered the predominant contributor to his presentation.

## Management

PCI of the LAD via the LIMA graft was performed. Given prior use of the radial artery as a bypass conduit, radial access was not feasible. A 6-F, 45-cm sheath (Terumo) was advanced via the right femoral artery, and a 6-F internal mammary guiding catheter (Medtronic) was used to engage the LIMA. The LIMA-to-LAD was wired with a Runthrough NS Extra Floppy guidewire (Terumo).

**Figure 1:**
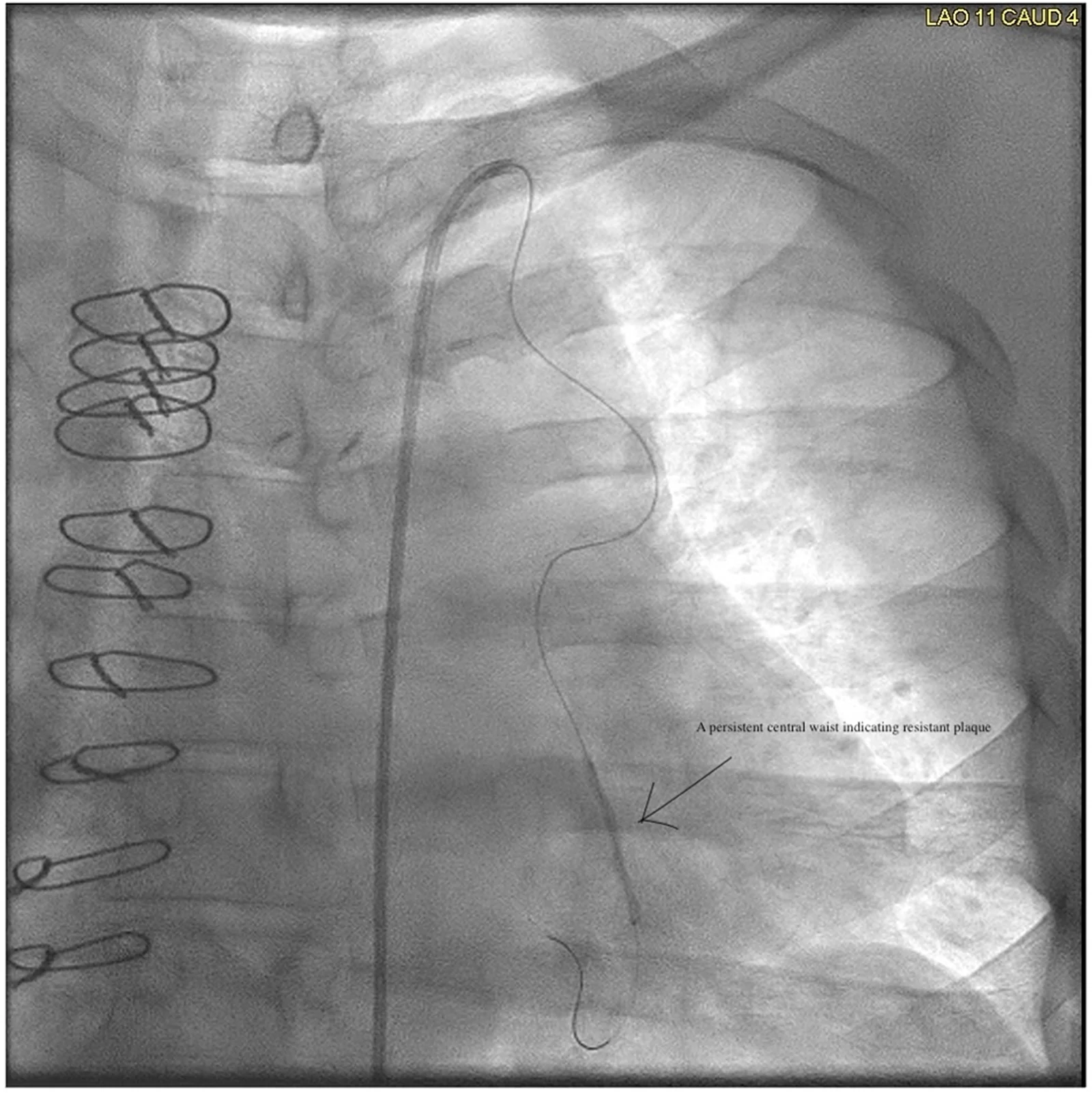
Noncompliant Balloon With Persistent Central Waist, Indicating Resistant Plaque Arrow shows a persistent central wait indicating resistant plaque.

The initial attempt to deliver an Emerge 2.5 × 20-mm semicompliant (SC) balloon (Boston Scientific) was unsuccessful because it could not be delivered to the lesion site. A Takeru 1.5 × 15-mm SC balloon (Terumo) was then able to be delivered to the lesion site, and inflated to 12 atm, allowing successful delivery of the Emerge 2.5-mm SC balloon, and inflated to 6 atm. Lesion preparation was further performed using an Emerge 2.0 × 30-mm noncompliant (NC) balloon (Boston Scientific), inflated to 20 atm. A persistent central waist was observed, indicating resistant plaque (Figure 1). The target lesion was approximately 10 mm in length and appeared predominantly fibrotic, with no significant angiographic calcification; however, resistance to high-pressure NC balloon inflation suggested underlying fibrocalcific plaque.

Given the inadequate lesion expansion, plaque modification was deemed necessary before stent implantation. A scoring balloon was selected for plaque modification given its effectiveness in resistant fibrotic lesions and its ability to facilitate plaque disruption. Alternative strategies such as cutting balloon angioplasty could also be considered; however, both scoring and cutting balloons carry a potential risk of graft trauma during delivery through the LIMA.

To minimize the risk of LIMA graft trauma during delivery of the scoring balloon and to provide additional support, an integrated microcatheter-guide extension system (CrossFast; Vantis Vascular) was advanced over the workhorse wire and carefully intubated into the distal LIMA up to the level of the LIMA-LAD anastomosis under fluoroscopic guidance. The inner microcatheter rail was subsequently removed while maintaining the guide extension in position to optimize device delivery (Videos 4 and 5). Continuous electrocardiographic and hemodynamic monitoring during deep intubation demonstrated no ischemic changes or pressure damping, confirming procedural stability.

Through the guide extension, a Scoreflex 2.0 × 10-mm NC scoring balloon (OrbusNeich Medical) was successfully delivered and inflated to 20 atm, resulting in full lesion expansion (Videos 6 and 7).

Intracoronary imaging was considered to guide lesion preparation and stent optimization; however, it was deferred because the imaging catheter could not be advanced through the integrated guide extension system because of size incompatibility. This represents a potential tradeoff of the system, balancing improved deliverability and graft protection against limitations in compatibility with certain intracoronary imaging modalities.

Subsequently, a Synergy XD 2.5 × 32-mm drug-eluting stent (Boston Scientific) was deployed in the mid-to-distal LAD and postdilated with an Emerge 2.5 × 20-mm NC balloon (Boston Scientific). Final angiography demonstrated a widely patent LIMA-to-LAD with no residual stenosis and TIMI flow grade 3 (Video 8).

## Outcome and Follow-Up

Final angiography demonstrated a patent LIMA-to-LAD with 0% residual stenosis and TIMI flow grade 3 in the native mid-to-distal LAD. The patient tolerated the procedure without complications.

At 1-month outpatient follow-up, the patient reported significant improvement in anginal symptoms, with only mild residual exertional chest discomfort, consistent with Canadian Cardiovascular Society angina class II. Given the angiographically severe stenosis of the PDA distal to the graft anastomosis, he was scheduled for staged PCI of the distal PDA at a later date.

## Discussion

PCI via the LIMA graft is technically challenging because of the graft’s length, tortuosity, and susceptibility to mechanical injury. Although LIMA grafts demonstrate excellent long-term patency, PCI through the LIMA remains relatively uncommon and is associated with unique procedural difficulties, including limited guide support and challenges with device delivery to lesions distal to the anastomosis. Expert opinion and case-based literature emphasize the need for careful catheter manipulation and thoughtful device selection when intervening via the LIMA.^1^

Iatrogenic injury to the LIMA graft during PCI, including dissection and perforation, has been described and may lead to catastrophic outcomes. Case reports have documented LIMA dissection occurring during PCI, underscoring the vulnerability of the conduit to mechanical stress.^2^ Similarly, LIMA graft perforation has been reported during complex interventions, highlighting that even contemporary interventional devices can cause significant graft trauma.^3^

Guide catheter extension systems are frequently used in complex PCI to improve coaxial alignment and facilitate device delivery. Observational studies have demonstrated their effectiveness but have also identified a low, yet clinically relevant incidence of device-related complications, including coronary dissection.^4^ Importantly, traumatic coronary injury related to guide extension catheter use has been described, reinforcing that deep intubation and aggressive manipulation may contribute to vessel injury, particularly in fragile conduits such as arterial grafts.^5^

Specialty balloons, including scoring balloons, are often required for plaque modification in resistant lesions. However, they pose additional challenges when advanced through a long and tortuous LIMA graft. The interaction between rigid scoring elements and the graft wall during delivery may further increase the risk of endothelial injury or dissection. However, strategies specifically aimed at mitigating graft trauma during delivery of specialty balloons via the LIMA are not fully described.

In this case, an integrated microcatheter-guide extension system was used not only to enhance support and facilitate delivery of a scoring balloon to the target lesion but also to serve as a protective conduit for the LIMA graft. By enhancing coaxial alignment and reducing the direct interaction between the scoring balloon and the graft wall during advancement, the system may help minimize mechanical stress on the vessel.

Unlike conventional guide extension systems, the integrated microcatheter-guide extension platform provides a lower crossing profile with improved trackability and deliverability, eliminating the need for balloon-assisted tracking. This design may reduce the risk of vessel trauma associated with advancing bulkier systems or using balloon anchoring techniques within fragile conduits such as the LIMA. In addition, the integrated system facilitates smoother distal delivery and more controlled deep intubation, which may be particularly advantageous in long and tortuous grafts.

This case highlights an underappreciated procedural risk—LIMA graft injury during specialty balloon delivery—and presents a practical strategy that may enhance procedural safety. As complex PCI via arterial grafts becomes increasingly encountered in contemporary practice, techniques that balance device deliverability with graft protection will be essential.

## Conclusions

PCI of the LAD via the LIMA graft is feasible but carries a risk of graft injury, particularly during delivery of specialty balloons. In this case, the use of an integrated microcatheter-guide extension system facilitated delivery of a scoring balloon while minimizing interaction with the LIMA graft. These findings represent a single-case experience and should be considered hypothesis-generating, warranting further investigation to assess safety and reproducibility.

**Take-Home Messages**

• Percutaneous coronary intervention via the LIMA graft carries a risk of graft trauma, particularly during delivery of specialty balloons such as scoring balloons.
• An integrated microcatheter-guide extension system may enhance device deliverability while providing a protective conduit for the LIMA graft during complex interventions.

### Declaration of generative AI and AI-assisted technologies in the writing process

Artificial intelligence tools were used only to assist with manuscript preparation. ChatGPT (version 5.4) was used to improve the readability of the manuscript, and Canva AI was used to assist in preparing the visual summary slide. These tools were not used to generate scientific content, data, or conclusions and did not substitute for human critical thinking or interpretation.

## Funding support and author disclosures

Dr Aronow has relationships with Amplitude Vascular Systems, Novartis, RapidAI, Medtronic, Recor, and Silk Road Medical. All other authors have reported that they have no relationships relevant to the contents of this paper to disclose.

## Appendix

Video 1 Patent LIMA Graft to the LAD With Severe Stenosis of the Native LAD, Distal to the LIMA Anastomosis LAD = left anterior descending artery; LIMA = left internal mammary artery.

Video 2 Patent Right Internal Mammary Artery Graft to the Right Coronary Artery With Severe Stenosis of the Native Posterior Descending Artery Distal to the Graft Anastomosis

Video 3 Patent Radial Artery Grafts to the Obtuse Marginal and Ramus Branch Arteries

Video 4 Delivery of an Integrated Microcatheter-Guide Extension System Over the Workhorse Wire to the Level of the Left Internal Mammary Artery-Left Anterior Descending Artery Anastomosis Under Fluoroscopic Guidance

Video 5 Removal of the Inner Microcatheter Rail While Maintaining the Guide Extension in Position

Video 6 Delivery of Scoring Balloon Over the Guidewire Through the Guide Extension

Video 7 Successful Inflation of a Scoring Balloon

Video 8 Final Angiogram Demonstrating a Widely Patent Left Internal Mammary Artery-to-Left Anterior Descending Artery With No Residual Stenosis and TIMI Flow Grade 3
